# A Systematic Review of Magnesium Sulfate for Perinatal Neuroprotection: What Have We Learnt From the Past Decade?

**DOI:** 10.3389/fneur.2020.00449

**Published:** 2020-05-27

**Authors:** Robert Galinsky, Justin M. Dean, Ingran Lingam, Nicola J. Robertson, Carina Mallard, Laura Bennet, Alistair J. Gunn

**Affiliations:** ^1^Department of Obstetrics and Gynecology, The Ritchie Centre, Hudson Institute of Medical Research, Monash University, Melbourne, VIC, Australia; ^2^Department of Physiology, University of Auckland, Auckland, New Zealand; ^3^Neonatology, Institute for Women's Health, University College London, London, United Kingdom; ^4^Department of Neuroscience and Physiology, University of Gothenburg, Sahlgrenska Academy, Gothenburg, Sweden

**Keywords:** neuroprotection, brain injury, hypoxic-ischemic encephalopathy, perinatal encephalopathy, magnesium sulfate, cerebral palsy

## Abstract

There is an important unmet need to improve long term outcomes of encephalopathy for preterm and term infants. Meta-analyses of large controlled trials suggest that maternal treatment with magnesium sulfate (MgSO_4_) is associated with a reduced risk of cerebral palsy and gross motor dysfunction after premature birth. However, to date, follow up to school age has found an apparent lack of long-term clinical benefit. Because of this inconsistency, it remains controversial whether MgSO_4_ offers sustained neuroprotection. We systematically reviewed preclinical and clinical studies reported from January 1 2010, to January 31 2020 to evaluate the most recent advances and knowledge gaps relating to the efficacy of MgSO_4_ for the treatment of perinatal brain injury. The outcomes of MgSO_4_ in preterm and term-equivalent animal models of perinatal encephalopathy were highly inconsistent between studies. None of the perinatal rodent studies that suggested benefit directly controlled body or brain temperature. The majority of the studies did not control for sex, study long term histological and functional outcomes or use pragmatic treatment regimens and many did not report controlling for potential study bias. Finally, most of the recent preterm or term human studies that tested the potential of MgSO_4_ for perinatal neuroprotection were relatively underpowered, but nevertheless, suggest that any improvements in neurodevelopment were at best modest or absent. On balance, these data suggest that further rigorous testing in translational preclinical models of perinatal encephalopathy is essential to ensure safety and best regimens for optimal preterm neuroprotection, and before further clinical trials of MgSO_4_ for perinatal encephalopathy at term are undertaken.

## Introduction

Perinatal encephalopathy is a significant public health issue. It is associated with high mortality, serious morbidity and significant costs. Most of this cost is related to long-term neurodevelopmental disabilities, such as cerebral palsy (CP). For example, in 2003 the combined lifetime economic cost of all cases of CP in the USA was over US$ 11.5 billion (1). This cost is largely attributed to the combined loss of productive members of society and the direct burden of care on the individual, family and social institutions, rather than medical costs.

Therapeutic hypothermia for moderate to severe neonatal encephalopathy (NE) in term and near-term infants improves survival without disability (2), but nearly half of all infants still died or survived with disability. Furthermore, infants born at <35 weeks of gestation are not currently candidates for therapeutic hypothermia, and so effective therapeutic interventions to improve long-term neurodevelopmental outcomes are needed (3, 4). The etiology of the long-term disability associated with prematurity is complex, and commonly includes factors such as exposure to both antenatal and postnatal infection/inflammation where therapeutic hypothermia may not be suitable (4).

Magnesium sulfate (MgSO_4_) is one of the most commonly used drugs in obstetric medicine. Its first reported use was in 1925 for control of eclamptic seizures (5). An early meta-analysis of MgSO_4_ for preventing threatened preterm labor showed it was ineffective at preventing preterm birth, and was associated with an increased risk of infant death (6). This analysis included studies of varied gestational ages, from <32 weeks up to 37 weeks, and included women that received a 4–6 g loading dose followed by a maintenance infusion of 1.5–6 g/hour intravenously or orally. More recent meta-analyses suggest that maternal MgSO_4_ is associated with a small but significant reduction in the risk of CP and motor dysfunction after premature birth (number needed to treat = 64), but it had no effect on overall death or disability (7, 8). These meta-analyses included women in labor or at high risk of labor at ≤ 33 weeks of gestation that received i.v. loading doses of 4–6 g of MgSO_4_ and/or a 1–3 g/hour maintenance dose or 5 g every 4 h IM. Based on these findings, MgSO_4_ is offered to mothers at high risk of preterm delivery in many countries (9).

Subsequently, there has been considerable interest in MgSO_4_ as a therapeutic intervention for preventing or mitigating the impact of perinatal brain injury (perinatal neuroprotection). In many western countries, MgSO_4_ is now a standard treatment in pregnancies threatened by preterm labor at <30 weeks' gestation. Furthermore, a clinical trial is currently in progress to assess whether giving antenatal MgSO_4_ to women at risk of preterm labor between 30 and 34 weeks of gestation improves outcomes at 2 years of age (ACTRN12611000491965) (10). MgSO_4_ is one of the cheapest options for potential neuroprotection and is associated with a relatively low risk of adverse effects, and so would be highly cost effective (11). Thus, its use in the developing and developed world would be highly justified, provided long-term benefit can be demonstrated.

Currently, two out of the five randomized controlled trials of maternal MgSO4 for threatened preterm labor from the original meta analyses have followed children up to school age and show no significant improvement in cognitive, behavioral, growth or functional outcomes, although these studies are relatively small due to incomplete follow-up (12, 13). Nevertheless, the lack of apparent long-term clinical benefit, even allowing for the smaller cohorts, raises the possibility that treating all pregnancies threatened by preterm labor may not be appropriate in the long-term. It is interesting to note that the exact mechanism/s by which MgSO_4_ may confer neuroprotection remains unclear (14). Thus, in order to elucidate which patient population may benefit from MgSO_4_ we must first carefully reflect on how it interacts with evolving brain injury.

The purpose of this review was to systematically evaluate preclinical and clinical studies on MgSO_4_ for perinatal neuroprotection (i.e., to reduce preterm and term brain injury) over the last decade and identify the most recent advances and knowledge gaps.

## Analysis Strategy

### Search Method

**Figure 1:**
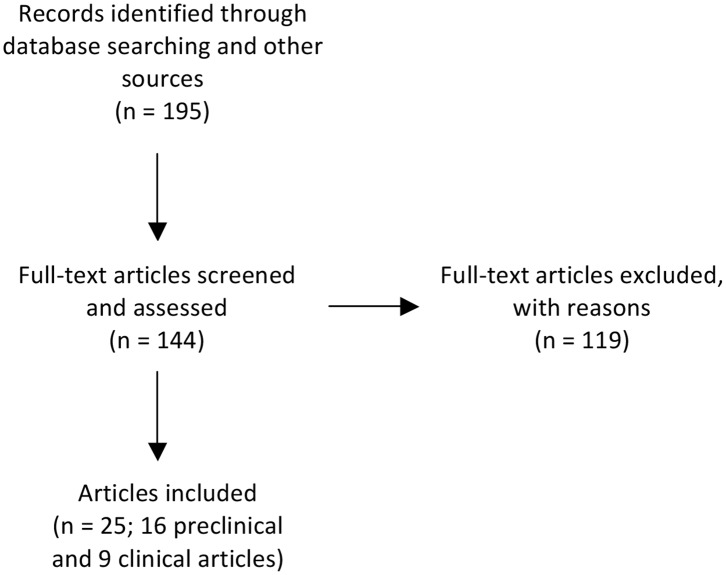
Flow chart illustrating the number of papers identified through database searching and other relevant sources, the number of full-text articles screened, assessed, and excluded, and the final number of original papers surveyed. Preclinical publications that performed ontogeny assessment of outcomes, used more than one paradigm of encephalopathy or multiple treatment timings were further sub-divided if outcomes differed according to age at assessment, experimental paradigm or treatment timing. After subdividing these publications, there was a revised total of 22 preclinical studies. For the purpose of reporting on the preclinical literature we have summarized the data based on the individual studies (*n* = 22).

In this review, a group of researchers focusing on perinatal neuroprotection examined preclinical (animal) and clinical (human) studies of MgSO_4_ for preterm and term neuroprotection in from January 1 2010, to March 1 2020 using the Preferred Reporting Items for Systematic Reviews and Meta-Analyses (PRISMA) guidelines for systematic review. Studies were searched for on Pubmed and Medline (OvidSP) using the following search terms: (preterm brain injury OR perinatal encephalopathy OR neonatal encephalopathy) AND (magnesium OR MgSO_4_). Other sources used to identify studies included relevant original manuscripts and reviews. Abstracts were initially identified and screened by an unbiased investigator (RG) from our study group and duplicated by another investigator (JD). Duplicate records identified from the different sources were removed (Figure 1). Full text articles were then obtained using Papers software (version 3.4.23; Labtiva Inc., Cambridge, MA, USA) for review and independent data extraction.

The studies were grouped into those that reported improved or unaffected/negative outcomes based on histological and/or functional assessments. Studies were further subdivided by species, age (preterm vs. term equivalent), type of insult to induce brain injury, timing of treatment (before and/or after the insult), extent of temperature monitoring (brain, core, ambient, or none), main study outcomes (histological, physiological and/or behavioral), survival time after treatment, sex and study bias.

### Selection Criteria

Selection of relevant studies was by group consensus. Preclinical studies were deemed to be eligible if they presented clear histological and/or functional outcomes from *in vivo* experiments. Histopathological outcomes were based on assessment of gray and/or white matter cell survival or tissue death. Human studies were eligible if they presented clear pathological or functional outcomes using MRI, ultrasound, CT or neurodevelopmental assessment.

Inclusion criteria for the preclinical and human studies were preterm or term equivalent *in vivo* studies. We excluded *in vitro* studies, those that did not meet the age criteria and paradigms of traumatic brain injury. Studies that were not designed to test the effect of MgSO_4_ for fetal or neonatal neuroprotection as a primary outcome measure were excluded to ensure that we screened the most relevant literature and were not reporting potential false positive or false negative information.

### Risk of Bias in Individual Studies

The methodological quality/risk of bias of the studies was assessed according to whether temperature was controlled, inclusion of males and females, insult and treatment were randomized and investigators were blinded to the intervention during histological/functional assessments. For human studies, additional quality criteria included whether the study was a prospective or observational and whether blinding was reported. The results are summarized in Tables 1–3.[Table T2]

**Table 1:** Studies of MgSO_4_ for preterm and term-equivalent neuroprotection reporting improved histological and/or functional outcomes.

**References**	**Species**	**Insult**	**Regime**	**Timing**	**Serum levels**	**T^**°**^**	**Pathology**	**Function**	**Survival**	**Sex**	**Random-ization/blinding**
(15)	Fetal rat, e20	Maternal LPS	270 mg/kg bolus + 27 mg/kg maintenance, + 270 mg/kg bolus, maternal s.c.	−2 to 2 h	?	Ambient: 37°C during the study, data not shown	↓ global expression of Casp-3, NF-KB, nNOS, IL-6 & TNF	None	4 h post LPS	?	✓/?
(16)	Fetal rat, e17	Maternal LPS	270 mg/kg bolus + 27 mg/kg maintenance + betamethasone s.c.	+30 min to +4 h	?	?	↑ % hippocampal NeuN staining, ↑ mRNA expression of MAP2, MBP in ♂LPS+MgSO_4_ vs. ♂LPS+vehicle	None	P 60	♀,♂	✓/?
(17)	Fetal rat, e18	Maternal LPS	270 mg/kg bolus, maternal s.c.	−2 to 2 h	?	Ambient: 37°C during the study, data not shown	MRI: ↓diffusivity and T2 intensities vs. control, No effect on tissue IL-1β protein expression.	None	P 25	♀	✓/?
(18)	Fetal rat, e16	Maternal LPS	270 mg/kg bolus + 27 mg/kg maintenance, + 270 mg/kg bolus, maternal s.c	−2 to 2 h	?	Ambient: 37°C during the study, data not shown	↓ brain phospho-nNOS, NF?B and CCL2 expression vs. LPS+vehicle	None	4 h post LPS	?	✓/?
(19)	Fetal rat, e18	Maternal LPS	270 mg/kg bolus + 27 mg/kg maintenance, + 270 mg/kg bolus, maternal s.c	−2 to +2 h	?	Ambient: 37°C during the study, data not shown	None	↑ learning and memory vs. LPS+vehicle	P 90	♂	✓/?
(20)	Fetal mice, e15	Maternal LPS	270 mg/kg bolus + 27 mg/kg maintenance, + 270 mg/kg bolus, maternal s.c	0 h	?	?	↓brain S100B protein expression vs. LPS+vehicle	None	4–6 h post LPS	?	✓/?
(21)	Fetal rat, e19	Bilateral UAO, 30 min	600mg/kg, maternal i.p.	−20 min	?	?	↓ global lipid peroxidation and ↑mitochondrial integrity score	None	60 min post LPS	?	✓/✓
(22)	Fetal rat, e19	Bilateral UAO, 20 min	600 mg/kg, maternal i.p.	−30 min	?	?	↓global lipid peroxidation vs. vehicle+UAO	None	30 min post LPS	?	?/?
(23)	Mouse, P5	HI, 8% O_2_, 45 min	600 mg/kg i.p.	−1 h	?	Ambient: 36°C during hypoxia, data not shown	↓ lesion area: 55% in HI+vehicle vs. 6% in MgSO_4_+HI, *p* <0.01	Improved motor function	5 d	♀,♂	?/?
(23)	Mouse, P5	HI, 8% O_2_, 45 min	600 mg/kg i.p.	−1 h	?	Ambient: 36°C during hypoxia, data not shown	Sub group analysis showed ↓ lesion area in the hippocampus and thalamus in ♂MgSO_4_+HI vs. ♂vehicle+HI	No improvements in motor or cognitive function	40 d	♀,♂	?/?
(24)	Rat, P6	HI: 6% O_2_, 60 min	100 mg/kg i.p.	−1 h	1.8 mmol/L	Ambient: 37°C during hypoxia, data not shown	↑MBP score, ↓microgliosis and apoptosis, no effect on oligodendrocyte survival	None	3–5 d	♂	?/✓
(25)	Rat, P4	HI+10% O_2_ 80 min	1.1 mg/g i.p.	−24 h	4.1 ± 0.2 mmol/L	Ambient: 36°C during hypoxia and 10 min recovery, data not shown	↓ %tissue loss in hippocampal and striatal gray and white matter	None	7 d	♀♂	✓/✓
(25)	Mouse, P5	HI+10% O_2_ 70 min	0.92 mg/g i.p.	−24 h	2.7 ± 0.3 mmol/L	Ambient: 36°C during hypoxia and 10 min recovery, data not shown	↓ % hippocampal tissue loss vs. vehicle+HI	None	7 d	♀♂	✓/✓
(26)	Rat, P7	HI+8% O2 60 min	1.1 mg/g i.p.	−6 d, −3 d, −1 d, −12 h,	3.3 mmol/L	Ambient: 36°C during hypoxia and 10 min recovery, data not shown	↓ global brain injury score and % tissue loss vs. vehicle+HI	None	7 d	♀♂	✓/✓
(27)	Fetal sheep, 0.85 g.a.	1 h partial UCO, then neonatal resuscitation	400 mg/kg, fetal i.v.	0 h	?	Core: 37–39°C, data not shown	↓ cortical and subcortical apoptosis, intracellular ROS and Ca2+ accumulation vs. vehicle + UCO	None	3 h	?	✓/✓

*CAO, Carotid artery occlusion; EEG, electroencephalography; e, embryonic day; g.a., gestational age; HI, hypoxia-ischemia; HT, hypothermia; LPS, lipopolysaccharide; MRS, magnetic resonance spectroscopy; P, postnatal day; T°, temperature; Tx, treatment; UAO, uterine artery occlusion; UCO, umbilical cord occlusion*.

**Table 2 T2:** Studies of MgSO4 for preterm and term-equivalent neuroprotection reporting no improvement in histological and/or functional outcomes.

**References**	**Species**	**Insult**	**Regime**	**Timing**	**Serum levels**	**T^**°**^**	**Pathology**	**Function**	**Survival**	**Sex**	**Random-ization/blinding**
(16)	Fetal rat e17	Maternal LPS	270 mg/kg bolus + 27 mg/kg maintenance + BM s.c.	+30 min – 4 h	?	?	None	No effect on motor function at P13 despite improvements at P5 and P9 in ♂LPS+MgSO_4_ vs. ♂LPS+vehicle	P 5, 9, 13	♀♂	✓/?
(20)	Fetal mice, e15	Maternal Mifepristone (RU486)	270 mg/kg bolus + 27 mg/kg maintenance, + 270 mg/kg bolus, maternal s.c	0 h	?	?	No change in S100B protein expression	None	4–6 h post RU486	?	✓/?
(28)	Fetal mice, e15	Maternal LPS	270 mg/kg bolus + 27 mg/kg maintenance, + 270 mg/kg bolus, maternal s.c	0 h	?	?	No effect on inflammatory, oligodendrocyte and astrocyte gene expression. ↑ Caspase-1 and IL-1β mRNA expression in LPS+MgSO_4_ vs. vehicle+MgSO_4_	None	6 h post LPS	?	✓/?
(26)	Rat, P7	HI+8% O_2_ 60 min	1.1 mg/g i.p.	−3 h, −30 min	3.3 mmol/L	Ambient: 36°C during hypoxia and 10 min recovery, data not shown	No differences between groups for injury score and % tissue loss vs. vehicle+HI	None	7 d	♀♂	✓/?
(26)	Rat, P7	HI+8% O2 60 min	1.1 mg/g i.p.	1 h	3.3 mmol/L	Ambient: 36°C during hypoxia and 10 min recovery, data not shown	No differences between groups for injury score and % tissue loss vs. vehicle+HI	None	7 d	♀♂	✓/✓
(29)	Fetal sheep 0.7 gestation	25 min UCO	160 mg loading, 48 mg/h/24 then 24 mg/h/24 h	−24–+24	1.9 ± 0.1 mmol/L	Brain: 39-39.6°C, continuous monitoring	↑oligodendrocyte loss. No effect on neuronal survival	↓seizure number and burden	3 d	♂♀	✓/✓
(30)	P1 piglets	Bilateral CAO+FiO2 6%	180 mg/kg loading then 8 mg/kg/h 48 h + HT vs. vehicle+HT	1 h	1.5 mmol/L	Core: normothermia: 38–39 vs. HT: 33.5–34°C, continuous monitoring	No regional improvements in cell death or oligodendrocyte survival. !!! ↓ in total cell death and ↑ oligodendrocyte survival in MgSO_4_+HT vs. vehicle+HT	No overall improvement in recovery of EEG or MRS markers of outcome!!! Secondary analysis suggested ↑ recovery of aEEG when mild cases were excluded	2 d	♂	✓/✓

*CAO, Carotid artery occlusion; EEG, electroencephalography; e, embryonic day; g.a, gestational age; HI, hypoxia ischemia; HT, hypothermia; LPS, lipopolysaccharide; MRS, magnetic resonance spectroscopy; P, postnatal day; T°, temperature; Tx, treatment; UAO, uterine artery occlusion; UCO, umbilical cord occlusion*.

**Table 3:** Human studies of MgSO4 for preterm and term/late preterm neuroprotection.

**References**	**Indication**	**Age at birth**	**Age at study**	**Timing**	**Pathology**	**Function**	**Sex**	**Study type**
(31)	Preterm neuroTx: chorioamnionitis	≤ 30 weeks, *n* = 228	2 y	Antenatal	No difference in rates of IVH between CA+MgSO4 vs. CA+placebo	No difference in rates of CP, mental or physical disability	✓	Prospective, double blinded multicenter RCT
(32)	Preterm neuroTx: chorioamnionitis	≥24 weeks *n* = 396	2 y	Antenatal	No difference in rates of IVH or PVL between CA+MgSO4 vs. CA+placebo	No difference in rate of stillbirth, death, moderate-severe CP or neurodevelopmental delay between groups	✓	Prospective, double blinded multicenter RCT
(33)	Preterm neuroTx	Mean ±SD: 28.3 ±2.2 weeks, *n* = 73	Mean ±SD: 32.4 ± 2 weeks	Antenatal	MRI: ↓ cerebellar hemorrhage in MgsO4 (*n* = 13/49) vs. control (*n* = 14/24), no effect on white matter injury or IVH	None	✓	Prospective, blinded single center study
(34)	Preterm neuroTx	Median ± SD: 27 ± 2, weeks, *n* = 64	24, 48, 72 h	Antenatal	Cranial ultrasound: ↓P/IVH in MgSO4 (*n* = 4/36) vs. vehicle (*n* = 9/28) at 72 h	NIRS: ↓cerebral O_2_ consumption vs. vehicle at 24 h	✓	Observational single center study
(13)	Preterm neuroTx	Mean ± SD: 27.3 ± 2.2, weeks *n* = 867	7–8 years old	Antenatal	None	No difference in cognitive, behavioral, growth or functional outcomes	✓	Prospective, double blinded multicenter RCT
(12)	Preterm neuroTx	27–32 weeks *n* = 501	7–14 years old	Antenatal	None	No significant improvement in motor dysfunction, behavior or cognition	✓	Prospective, double blinded multicenter RCT
(35)	Preterm neuroTx	24–32 weeks, *n* = 475	Hospital discharge	Antenatal	No difference in rates of IVH or PVL. Secondary outcomes showed higher incidence of ROP. Subgroup analysis showed higher neonatal mortality rate with increasing magnesium levels	MgSO_4_ group took longer to reach full feeds and had greater length of hospital stay vs. placebo	✓	Retrospective study
(36)	Term HIE	≥35 weeks *n* = 60	Not stated, assessed at hospital discharge	≤ 6 h after birth	No difference in HIE severity, intracranial hemorrhage or death between MgSO_4_+HT (*n* = 29) vs. vehicle+HT (*n* = 31)	No difference in seizures	χ	Prospective, double blinded multicenter RCT
(37)	Term HIE	38–39 weeks, *n* = 32	Follow up to 6 months	≤ 30 min, 24 and 48 h after birth	Brian CT: No difference in severity of HIE in MgSO_4_ (*n* = 16) vs. control (*n* = 16)	No difference in incidence of death, seizures, discontinuous EEG or neurodevelopmental assessments at discharge and 6 months	χ	Prospective single center RCT

*CA, chorioamnionitis; CP, cerebral palsy; CT, computed tomography; EEG, electroencephalography; HIE, hypoxic ischemic encephalopathy; P/IVH, peri/intraventricular hemorrhage; NIRS, near infrared spectroscopy; PVL, periventricular leukomalacia; RCT, randomized controlled trial; Tx, treatment*.

## Results

We identified 195 records. After excluding reviews and records for which a full text was not available, we screened a total of 144 full text articles. One hundred and nineteen were excluded due to one or more of the following: inappropriate developmental age, *ex vivo* studies, histological and/or behavioral outcomes were not examined, MgSO_4_ was not used for fetal or neonatal neuroprotection or assessment of MgSO_4_ for fetal or neonatal neuroprotection was a secondary outcome measure. Thus, a total of 25 individual publications, consisting of 16 preclinical and 9 clinical publications, were included in this analysis (Figure 1). Preclinical publications that performed ontogeny assessment of outcomes, used more than one paradigm of encephalopathy or multiple treatment timings were further sub-divided if outcomes differed according to age at assessment, experimental paradigm or treatment timing. After subdividing these publications, there was a revised total of 22 preclinical studies. For the purpose of reporting on the preclinical literature we have summarized the data based on the individual studies (*n* = 22). Studies were then stratified by species (rodent, large animals, human), fetal or postnatal intervention, and outcome (neuroprotection or no neuroprotection). For clarity, rodent studies that used an antenatal intervention are referred to as “fetal rodent studies,” whereas rodent studies that used a postnatal intervention are referred to as “postnatal rodent studies.”

### Preclinical Studies of MgSO_4_ for Perinatal Neuroprotection

Most of the fetal rodent studies used acute maternal LPS injection, leading to a peak maternal systemic inflammatory response within ~6 h (38). All of the postnatal rodent studies used the Rice-Vannucci model of unilateral carotid artery ligation followed by a period of moderate hypoxia (39). This functional paradigm of perinatal brain injury is associated with infarction of the middle cerebral artery region ipsilateral to the ligated carotid artery, with no overt cell death in the contralateral cerebral hemisphere. In sheep, cerebral injury was caused by either partial umbilical cord occlusion or complete umbilical cord occlusion, leading to either a basal ganglion-predominant or a watershed pattern, as previously reviewed (40). Similar regional brain injury is observed in neonatal piglets following hypoxia ischemia (HI; cerebral hypoperfusion with hypoxia) (41, 42).

Half of the studies (11/22) used fetal rats or mice (e15-20), 3/11 assessed outcomes postnatally (16, 17, 19). These gestational ages at the time of the insult broadly correspond to the neural development of human infants at <24 weeks of gestation (43, 44). Most of these studies measured markers of neuroinflammation and apoptosis, one study measured injury based on tissue S100B expression, one study used diffusion weighted and T2 magnetic resonance imaging (MRI) to broadly assess microstructural pathology, and 2 studies assessed behavior for motor or cognitive performance. Four studies quantified global expression of markers related to white matter integrity, inflammation or oxidative stress, none performed quantitative assessment of oligodendrocyte survival or myelin density. Most of the fetal rodent studies used maternal LPS to induce encephalopathy. Two fetal rat studies used bilateral uterine artery occlusion and one used mifepristone (RU486) as a model of non-inflammation associated preterm birth.

Eight out of 22 studies (36%) used postnatal rodents and induced injury using the Rice-Vannucci model. Five studied rats at P3–P6, which is broadly equivalent to the preterm human brain at 24–32 weeks of gestation, and 3 studied rats at P7 which is comparable to the preterm human at 30–32 weeks of gestation (43, 44). Most of the postnatal rodent studies examined infarct area to assess neuronal loss/survival/severity of the insult, one quantified oligodendrocyte survival and 2 assessed behavior for motor and cognitive function.

Three out of 22 studies (14%) used large animals. In the large animal studies, fetal sheep were exposed to global hypoxia via umbilical cord occlusion, whereas piglets received bilateral carotid artery occlusion combined with inhalational hypoxia. One study used fetal sheep at 0.8 (120 out of 147 days) of gestation and one used 1 day old piglets; both paradigms are comparable to the term human (45, 46). A separate study used fetal sheep at 0.7 (104 out of 147 days) of gestation, which is equivalent to human brain development at ~30 weeks of gestation (47). Histological outcomes were combined with assessment of neuronal functional and seizures in preterm fetal sheep and term piglets using electroencephalography. The term piglet study also used magnetic resonance spectroscopy to assess brain energy metabolism as one of the primary outcome measures.

### Regime and Timing of Magnesium Delivery

Most of the rodent studies that used a fetal intervention (fetal rodent studies; 10/11) administered MgSO_4_ to the pregnant dam subcutaneously using a 270 mg/kg bolus followed by a 27 mg/kg maintenance infusion every 20 min for 4 h. An additional 270 mg/kg MgSO_4_ bolus was given at the end of the 4 h period in all but 1 study, in which MgSO_4_ and betamethasone were given 30 min after maternal lipopolysaccharide (LPS) injection. However, in that study the effect of MgSO_4_ alone was not tested. In postnatal rodents, MgSO_4_ was administered s.c. or i.p. as a single dose that ranged from 100 to 1,000 mg/kg. In the large animal studies the dose ranged from 160 to 400 mg/kg and was delivered intravenously. Two out of 3 large animal studies (1 preterm fetal sheep and 1 term piglet paper) used an additional maintenance infusion over 48 h (range: 16–48 mg/kg). The majority of the studies (14/22; 64%) administered MgSO_4_ before the insult (−24 h to −30 min), 12/14 reported that MgSO_4_ was associated with neuroprotection. Six out of 22 studies (27%) started treatment immediately after the insult (≤30 min), 3/6 reported MgSO_4_ was associated with neuroprotection. Two out of 22 studies (9%) delayed treatment to 1 h post insult, neither of these studies reported significant improvements in histological and/or functional outcomes.

### Neurological Outcome and Survival Time After Injury

Fifteen out of the 22 perinatal studies (68%) reported improved neural outcomes with MgSO_4_ treatment. One of these studies reported improved outcomes when MgSO_4_ was combined with betamethasone in the setting of maternal inflammation, although the effects of MgSO_4_ alone were not assessed. Intriguingly, in sham controls combination therapy was associated with loss of NeuN immunoreactivity, indicating reduced neuronal survival (16). Eleven out of 15 studies (73%) used relatively short survival times of <1 d (*n* = 6) or 1–7 d (*n* = 5). One out of 15 studies (7%) examined outcomes after 2–4 weeks. Three out of 15 studies (20%) used survival times of 5–13 weeks. Two out of 3 studies, that used survival times of 35 and 64 days, showed histological injury and improvements with treatment were limited to males (there were no long-term effects of the insult or treatment in females) (16, 23), however there were no improvements in motor or cognitive function in male offspring (23). One study, which was limited to male offspring, reported improvements in learning and memory after 93 days but did not examine histology (19).

Seven out of 22 studies (32%) reported no neuroprotection or deleterious effects associated with MgSO_4_ treatment. One study in fetal rodents reported no effect of MgSO_4_ on expression of pyroptotic and pro-inflammatory genes after maternal LPS exposure, or on pro-oligodendrocyte and glial markers. However, MgSO_4_ treatment plus LPS was associated with increased expression of pyroptotic and pro-inflammatory gene expression compared to sham controls (28). One study assessed combination therapy with MgSO_4_ and therapeutic hypothermia in term piglets exposed to HI and showed reduced total numbers of TUNEL positive cells and increased total numbers of oligodendrocytes compared to therapeutic hypothermia alone. However, MgSO_4_ was not associated with any improvements in regional cell survival or in magnetic resonance spectroscopy measures of outcome at 48 h compared with hypothermia. One study in preterm fetal sheep exposed to severe asphyxia reported reduced seizures but no improvement in regional neuronal survival and reduced survival of oligodendrocytes in MgSO_4_ treated fetuses compared to vehicle+asphyxia. One study in rodents exposed to maternal LPS reported improvements in motor function at P5 and P9, however at P13 there was no effect of treatment (16). Six out of 7 studies used relatively short survival times of <1 d (*n* = 2) or 1–7 d (*n* = 4). One study examined outcomes after 2 weeks.

### Temperature Monitoring

In the fetal rodent studies that reported improved outcomes with MgSO_4_, 4/8 studies controlled ambient temperature and 4 did not report temperature monitoring in their experimental protocol. All 6 postnatal rodent studies that reported improved outcomes with MgSO_4_ monitored ambient temperature. However, 3/6 monitored ambient temperature during 10 min of recovery and 3/6 monitored temperature during HI but not the recovery period. One study in preterm neonatal lambs that showed improved outcomes with MgSO_4_ reported control of core temperature during the study period but data were not shown. Furthermore, the core temperature range reported in this study was 37–39°C. Critically, the lower end of this range is ~2–3°C lower than the average core temperature of a neonatal lamb.

Three out of 7 studies that showed no improvement or deleterious effects with MgSO_4_ did not report temperature monitoring; all used rodents exposed to antenatal insults and treatment. Two studies, both in the P7 rat, reported ambient temperature control during the first 10 min of recovery. All of the large animal studies that showed no improvement/some modest improvements/deleterious effects with MgSO_4_ (2/7) reported continuous core or brain temperature data throughout the study period.

### Sex

Six out of 15 studies (40%) that reported improved outcomes used both sexes. One of these studies in postnatal rodents exposed to HI reported histological benefits were limited to MgSO_4_-treated males but no improvements were seen in motor and cognitive deficits. Two studies only used males, and one study only used females. Six studies did not report the sex of the subjects.

Four out of 7 studies that reported no improvement in outcomes used both sexes. One study in neonatal piglets examined males. Two studies did not report the sex of the subjects.

### Measurement of Magnesium in the Circulation and Brain

Seven out of 22 studies reported circulating magnesium concentrations in their subjects. Levels in small and large animal studies ranged from 1.5 to 4.1 mmol/L (100–350% increase from baseline). One study in piglets reported a 16 % increase in CSF magnesium levels during treatment.

### Study Bias

Five out of 15 studies (33%) that showed improved outcomes reported subjects were randomized to the insult and treatment, and blinding of assessors. Six out of 15 studies (40%) reported randomization but did not report blinding of assessors. One out of 15 studies (7%) reported blinding but not randomization of subjects to the insult or treatment. Three out of 15 studies (20%) did not report randomization or blinding as part of their experimental protocol.

Four out of 7 studies (57%) that showed no improvement/some modest improvements/deleterious effects reported randomization of subjects to the insult and treatment, and blinding of assessors during analyses. Three out of 7 (43%) studies reported randomization of subjects to the treatment and insult but did not report blinding of assessors in their protocol.

### Human Publications

We identified 9 human publications. Two papers were follow-up analyses of large randomized controlled trials investigating school aged outcomes in preterm infants treated with antenatal MgSO_4_ (*n* = 503–669) (12, 13). Two papers were secondary analyses of a randomized control trial of MgSO_4_ for fetal neuroprotection (*n* = 228–396) (31, 32). Five papers reported short term outcomes in preterm or term patients; 4 papers assessed a relatively small number of patients (total *n* = 32–71) (33, 34, 36, 37), while 1 assessed short term outcomes from a larger population (*n* = 475) (35).

Seven papers examined preterm infants that received antenatal MgSO_4_ for fetal neuroprotection. Two papers reported no significant improvement in cognitive, motor, behavioral, growth or functional outcomes in school age children (12, 13). Two were sub-group analyses focusing on infants exposed to clinical chorioamnionitis from a large randomized controlled trial (48); both showed MgSO_4_ was not associated with improved neurodevelopment at 2 years of age or reduced rates of intraventricular hemorrhage (IVH) or periventricular leukomalacia (PVL) (31, 32). Two papers reported a reduction in cerebral or cerebellar hemorrhage in the MgSO_4_ group vs. placebo, however rates of hemorrhage were low and the total number of patients included was relatively small (*n* = 64–71) (33, 34). One publication in 475 preterm infants reported no effect of MgSO_4_ on rates of IVH and PVL at hospital discharge. Secondary analyses showed higher rates of retinopathy of prematurity, longer time to reach full feeds and a higher length of hospital stay in the MgSO_4_ group vs. placebo (35). Two papers examined term/near term infants; one used postnatal MgSO_4_ as an adjunct to hypothermia (36), the other used MgSO_4_ alone (37). Treatment was administered within the first 30 min or 6 h after birth. There were no differences in pathological or functional outcomes between groups in both of the term human studies which were assessed at hospital discharge and 6 months of age. Neurological outcomes from the human studies were not consistent across the studies and so were not able to be compared.

Seven out of 9 papers (all preterm) reported equal ratios of males and females in their demographic data (12, 13, 31–35). Two studies, both in term infants, did not report patient sex (36, 37). Seven out of 9 papers were prospective (12, 13, 31–33, 36, 37), one study in preterm infants was an observational analysis (34) and 1 was a retrospective analysis (35). Two out of 9 studies (1 preterm and 1 term) did not report blinding in their study protocol (34, 37).

## Discussion

This systematic review shows that the effect of MgSO_4_ for neuroprotection in preterm and term-equivalent models of perinatal encephalopathy was highly inconsistent between studies in the last decade. Of particular concern, none of the perinatal rodent studies that suggested benefit directly controlled body or brain temperature or delay therapeutic intervention beyond the first hour after the experimental insult, whereas studies that did report control of core or brain temperature showed either limited or no improvement in outcomes. The majority of the studies did not control for sex or study long term histological and functional outcomes and many did not report appropriate measures to control for potential study bias. Moreover, although many of the recent preterm or term equivalent human studies that tested the potential of MgSO_4_ for perinatal neuroprotection were relatively small and likely to be underpowered, none report significant improvements in neurodevelopment, and follow up studies of the original multicenter randomized controlled trials of maternal MgSO_4_ for neuroprotection suggest that MgSO_4_ is not associated with improved outcomes at school age.

### Iatrogenic Hypothermia

As previously reviewed (14), magnesium consistently promotes peripheral vasodilation in rodents, large animals and humans (49–51). Thus, a magnesium-induced increase in cutaneous perfusion is likely to increase heat loss through radiation (50); this may be considerable in small animals with a relatively large surface area and low thermal stability (52). Supporting this concept, studies in adult rodents showed that neuroprotective effects of MgSO_4_ were associated with mild hypothermia and that neuroprotective effects were abolished if normothermia was maintained. In the preclinical studies surveyed here, over half (53%) of those that reported neuroprotection with MgSO_4_ used maternal LPS exposure to model antenatal infection/inflammation. Maternal LPS exposure commonly leads to maternal pyrexia (53), however, none of these studies reported maternal body temperature. Critically, intrapartum fever is associated with adverse neonatal outcomes (54–56) and increased risk of unexplained cerebral palsy or neonatal encephalopathy (57). Furthermore, it is well-established that mild hyperthermia of just 1 to 2°C can worsen neural injury (58, 59), possibly via greater release of oxygen free radicals and excitatory neurotransmitters, enhanced toxicity of glutamate on neurons, greater dysfunction of the blood brain barrier and proteolysis (40, 60). Half of the studies that used maternal LPS to study MgSO_4_ for fetal neuroprotection did not report temperature monitoring in their study protocol, while the remaining studies reported ambient temperature monitoring during the study; none presented temperature data in their results.

Most of the remaining studies used postnatal rodents (P5–P7) exposed to HI to evaluate MgSO_4_ for neonatal neuroprotection. All reported maintenance of ambient temperature during hypoxia, while three studies reported maintenance of ambient temperature during the first 10 min of recovery. This is an important consideration because even healthy postnatal rodents show fluctuating body temperatures in typical laboratory conditions between P5 and P14 (61). As previously reviewed, core temperature in postnatal rodents will be affected by the amount and composition of nesting material, position in the nest, huddling, distance from the dam and time since the last period of suckling (62). Unlike these studies of neonatal treatment, in the study of preconditioning with MgSO_4_, HI was induced 24 h after MgSO_4_ administration, at a time when magnesium levels had returned to normal and so vasodilation presumptively will have resolved (26). By contrast, one of the two large animal translational models of perinatal brain injury in which body or brain temperature was directly controlled suggested no effect after 2 or 3 days of recovery (29). The other study, in term piglets, reported more rapid recovery of aEEG after excluding mild cases, and a modest improvement in total cell death and numbers of surviving oligodendrocytes (30). Collectively, these data suggest that significant intrapartum/age related and drug induced fluctuations in body temperature, which can affect the severity of neural injury and confound preclinical studies of MgSO_4_ for perinatal neuroprotection, could have been missed in the majority of recent preclinical studies. Thus, irrespective of the experimental paradigm or species used, careful control of brain or body temperature is essential to ensure that outcomes are not confounded by iatrogenic hypothermia.

### Survival Time

One of the major limitations of the perinatal studies identified in this review is that most studies used relatively short survival times. For example, 17/22 preclinical studies (77%) used survival times that ranged from 30 min to 7 days after the insult. Although these shorter survival times provide important information about acute histological/functional outcomes, it is well-established that injury evolves over many weeks to months after the insult (4). Only three studies identified in this review assessed neurodevelopment after 35 to 90 days. All studied rodents and reported improvements in neurodevelopment after treatment. Two studies reported sex specific improvements with treatment in male offspring. However, one study was limited to semi-quantitative assessment of histology and gene (mRNA) expression in P60 rats exposed to antenatal inflammation that were not coupled with functional outcomes and 1 study reported histological improvements at P40 after HI that were not associated with improvements in motor or cognitive function (16, 23). Another study limited to male offspring showed cognitive improvements at P90 after exposure to antenatal inflammation, however histological analyses were not reported (19).

Consistent with the limited evidence for long-term benefits in preclinical studies, school aged outcomes of very preterm infants after antenatal treatment with MgSO_4_ show no significant improvements in cognitive, behavioral, growth or functional outcomes, although these studies are limited by incomplete follow-up (12, 13). Thus, further methodologically sound preclinical studies focusing on the long-term histological and functional impacts of MgSO_4_ are needed.

### Sex

Over half of the preclinical studies surveyed (12/22; 55%) in this review reported outcomes for only one sex or did not specify the sex of the subjects used. Preclinical and clinical studies have reported sexual dimorphisms in the severity and evolution of perinatal encephalopathy and in responses to treatment, as previously reviewed (62–64). Although randomized controlled trials of antenatal MgSO_4_ have not reported sex related differences in neonatal outcomes (13), recent preclinical studies in small and large animals have found sexual dimorphisms relating to functional and histological outcomes with MgSO_4_. In preterm fetal sheep, MgSO_4_-treated males showed greater suppression of neuronal excitation compared to females, as shown by greater loss of high frequency neural activity and increased suppression of seizures during the secondary phase of HIE (65, 66). Similarly, we identified two rodent studies that showed long term sex specific differences in histological and functional outcomes associated with MgSO_4_ (16, 23). Critically, two of the human trials identified in this review did not report patient sex in their demographic data (34, 37). Overall, these data raise the possibility that sex-specific effects of MgSO_4_ may arise in preterm infants and should be considered in future preclinical and clinical investigations.

### Bioavailability

One of the great unknowns associated with MgSO_4_ for perinatal neuroprotection is whether direct or maternal administration can sufficiently raise brain and CSF concentrations of magnesium to the level required for suppression of neural metabolism/neuroprotection. A key potential benefit of MgSO_4_ relates to its capacity to modulate N-methyl-D-Aspartate (NMDA)-glutamate mediated excitotoxicity (67). *In vitro* studies show that magnesium alleviates excitotoxic damage by binding to the magnesium site on the NMDA glutamate channel to reduce the accumulation of cytotoxic levels of intracellular calcium (68). In this setting, increased extracellular magnesium is associated with recovery of high energy phosphate stores, improved rates of protein synthesis and neural preservation in ischemic hippocampal neurons from fetal and adult rodents (69, 70), and after global ischemia in adults when magnesium was given by direct intra-hippocampal injection (71). However, benefit required a 2–4-fold increase in extracellular magnesium concentrations. It is well-established that hypermagnesemia is associated with potentially deleterious effects, such as respiratory depression and increased requirement for assisted ventilation (72). This is an important consideration since in preterm lambs and humans, mechanical ventilation is associated with neural injury (4, 73).

Until recently, it has been unclear whether it is possible to achieve such concentrations *in vivo* after peripheral administration. In preterm fetal sheep increasing the plasma magnesium concentration to levels that were comparable to those measured in human neonates was associated with a reduction in EEG activity and significant anticonvulsant effects after asphyxia, but did not reduce diffuse white matter injury or subcortical neuronal loss 3 days after the insult (29). Pragmatically, these data show that a clinically comparable increase in serum magnesium levels is sufficient to produce a central anti-excitatory effect but does not improve acute pathological outcomes.

Consistent with these data, in preterm neonates exposed to antenatal MgSO_4_, reduced cerebral oxygen consumption was reported during the first 24 h after birth (34). Furthermore, studies in term equivalent neonatal piglets exposed to HI showed that a 2-fold increase in circulating magnesium concentration was associated with only a modest rise (16%) in magnesium concentration within the cerebrospinal fluid, that did not augment hypothermic neuroprotection (30). These data are consistent with previous reports in adult dogs and rats, whereby a 3- to 4-fold increase in circulating magnesium levels produced a relatively small increase in magnesium levels within the CSF and parenchyma (74, 75). Nevertheless, a clinically comparable increase in circulating magnesium levels does modulate neural excitation, but does not seem to be sufficient to achieve neuroprotection.

### Treatment Timing and Population

A key translational consideration for testing neuroprotectants is when to treat. The majority of preclinical studies surveyed (20/22; 91%) started the intervention before the insult or immediately after the insult (within the first 30 min). Just 2 out of 22 studies initiated treatment within the first hour after the insult, one showed MgSO_4_ was not associated with neuroprotection, while the other suggested a modest improvement. Scientifically, the mechanisms of intra-insult cell death, particularly in the setting of HI, are different from those after treatment. Furthermore, it is well-established that inflammation (sterile or infective) is a key contributor to perinatal encephalopathy and is an important target for any neuroprotective intervention, particularly delayed therapy (4). Therefore, efficacy during pretreatment does not necessarily indicate the treatment will be effective if the insult has already started to evolve.

In an elegant study by Koning et al., pretreatment with MgSO_4_ from 6 days to 12 h before HI in P7 rats was associated with reduced oxidative stress and inflammation, and reduced severity of neural injury, likely due to preconditioning of mitochondrial resistance (26). As previously reviewed (4, 76, 77), preclinical and clinical studies have shown that antenatal preconditioning is associated with improved tolerance to subsequent insults (78–82). Conversely, preconditioning can induce sensitization to subsequent insults (78, 83, 84). Thus, translating preconditioning into improved outcomes is complex and requires careful further investigation before it can be applied in practice.

Clinically it is difficult to identify fetuses who are at risk of developing encephalopathy in advance, since the positive predictive value of fetal heart rate monitoring for predicting adverse neurodevelopmental outcomes is low (85). Furthermore, it remains a formidable challenge to start any intervention within the first 4–5 h after birth (2). None of the human studies surveyed reported a beneficial effect of MgSO_4_ on neurodevelopment after antenatal treatment, two of these studies were sub-analyses of a randomized controlled trial that focused on infants exposed to clinical chorioamnionits (31, 32). Two relatively small studies in preterm infants that received antenatal MgSO_4_ reported a reduction in the incidence of cerebral or cerebellar hemorrhage, however, neurodevelopmental outcomes were not reported (33, 34). Postnatal treatment (30 min to 6 h) with MgSO_4_ alone or as an adjuvant to therapeutic hypothermia did not improve short-term outcomes in term infants with HIE, however, both studies were based on relatively small cohorts and one of these trials is ongoing (The MagCool Study; NCT01646619) (36, 37).

These data are broadly consistent with data from a study in piglets that suggests MgSO_4_ was associated with only modest improvement in histological outcomes with no effect on magnetic resonance spectroscopy markers of long-term outcomes (30). This suggests that more pre-clinical research is essential before large multicenter RCTs of MgSO_4_ for neonatal encephalopathy at term should be considered. Furthermore, three of the preclinical studies surveyed reported negative effects of MgSO_4_ on cell survival and oligodendrocyte development (16, 28, 29), and one clinical study reported higher cord blood magnesium levels were associated with an increased risk of neonatal mortality, thus highlighting potential safety considerations. Collectively, these data indicate that we must improve our understanding of the optimal treatment regimen and patient population that are most likely to demonstrate benefit from MgSO_4_, before progressing further down the translational pipeline.

### Bias and Methodological Quality

Over half of the preclinical studies surveyed (13/22; 59%) in this review reported only randomization of subjects to the insult and treatment or blinding of assessors, or did not document either method in their study protocol. Most of these studies (10/13; 77%) reported neuroprotection with treatment. We found that all of the rodent studies that associated MgSO_4_ with neuroprotection did not perform brain or body temperature monitoring, allowing for neuroprotection to be associated with confounding mild hypothermia rather than MgSO_4_ *per se*. We identified one large animal study that reported core temperature monitoring in their experimental protocol and improved histological outcomes with MgSO_4_, however, continuous data were not shown and the lower end of the core temperature range was ~2–3°C lower than normal core temperature (27). By contrast, one of the two large animal studies that reported stable continuous monitoring of brain or body temperature showed no significant improvements in histological and functional outcomes (29). The other large animal piglet study with continuous monitoring of body temperature did show some modest improvement in overall oligodendrocyte survival, although not in magnetic resonance spectroscopy measures of outcome (30). These data highlight some inconsistencies in the methodological quality of the studies surveyed. It is not possible to know if the methodological issues identified in our analysis affected the outcomes of the studies. However, if this critical information is not reported in the publications it makes it difficult to assess the significance of past and future studies in a meaningful way.

## Conclusion

The majority of recent studies of MgSO_4_ for perinatal neuroprotection identified in this systematic review did not report measuring or maintaining body or brain temperature. In addition, most of the studies did not address pragmatic treatment regimens or the effect of different timings for initiation and duration of treatment, report or test the effect of the sex of the subjects, assess long-term functional and histological outcomes or report randomization and blinding of assessors in their study protocol. Furthermore, although the original meta analyses demonstrate that MgSO_4_ is associated with a reduction in the risk of cerebral palsy at 2 years of age (7, 8), the recent follow up studies, whilst relatively small, do not suggest a clear benefit at school age (12, 13). Additionally, many of the clinical and preclinical studies did not report whether they were adequately powered to detect differences in primary outcome measures between groups. In large animal and human trials, short-term analyses of MgSO_4_ for HIE at term do not indicate significant improvements in functional measures of outcome, and only one study in piglets suggested a modest effect on white matter histology. Taken collectively, these observations suggest that further pre-clinical testing is needed to determine possible differential effects of MgSO_4_ in males and females, whether there is a specific effect of MgSO_4_ on oligodendrocytes, and long-term effects on functional outcomes to ensure safety and to identify the optimal regimens for neuroprotection.

## Author Contributions

RG and AG conceptualized and designed the study. RG performed the initial search and drafted the paper. JD undertook replication of the search. RG, JD, IL, NR, CM, LB, and AG provided in depth review of the studies identified in the search, revised the paper, critically reviewed the paper, approved the final version as submitted, and agreed to be accountable for all aspects of the work.

## Conflict of Interest

The authors declare that the research was conducted in the absence of any commercial or financial relationships that could be construed as a potential conflict of interest.
